# Turning 75: Why Age Matters for Victims of Assaultive Violence Reported to Police

**DOI:** 10.1093/geroni/igaa057.155

**Published:** 2020-12-16

**Authors:** Lynn Addington

**Affiliations:** American University, Washington, District of Columbia, United States

## Abstract

Americans are able to age in more active and engaged ways than previous generations. These changes bring many positive opportunities, but also might affect the risk of criminal victimization for older adults. In considering these risks, an initial question is how best to identify older adults. One common default is to use age 65 and older, which suggests older individuals are part of a homogenous group. The Census Bureau’s multiple category approach illustrates another option, which captures variations as Americans age. This study explores the risk and characteristics of non-fatal assaultive violence using a multiple-category age definition and uses police data from the Federal Bureau of Investigation’s 2016 National Incident-Based Reporting System (NIBRS). These data were collected from 34 states and cover a range of crimes including aggravated and simple assaults. NIBRS data are well suited for this study as they collect details about the crime including victim and offender demographics and incident details such as victim-offender relationship, weapons, location and arrest. Preliminary results indicate that 34,689 assault victims were adults over the age of 65. Using a generic measure of older adult (age 65 and above) masks important variations in these assaults. Distinct patterns are observed between those aged 65 to 74, 75 to 84 and 85 and above. Within these age categories, differences also occur across racial and sex groups. The patterns observed can provide more nuanced guidance to challenge traditional assumptions about older adult crime victims and inform policies tailored to support these victims.

